# Hemoglobin S polymerization and sickle cell disease: A retrospective on the occasion of the 70th anniversary of Pauling's *Science* paper

**DOI:** 10.1002/ajh.25687

**Published:** 2019-12-31

**Authors:** William A. Eaton

**Affiliations:** ^1^ Laboratory of Chemical Physics National Institute of Diabetes and Digestive and Kidney Diseases, National Institutes of Health Bethesda Maryland

## Abstract

70 years ago, Linus Pauling, the legendary genius of 20^th^ century chemistry, published his famous work on the molecular cause of sickle cell disease, a paper that gave birth to what is now called molecular medicine. In this paper, Pauling left important questions unanswered that have motivated an enormous amount of scientific and clinical research since then. This retrospective discusses the basic science studies that have answered those questions directly related to the kinetics and thermodynamics of hemoglobin S polymerization.

## 1 INTRODUCTION

In this retrospective on the occasion of Pauling's 1949 *Science* paper,^1^ I present the major findings on the thermodynamics, kinetics, and mechanism of hemoglobin S polymerization. And, I describe how these basic science studies have led to a deeper understanding of the molecular pathogenesis of the disease and strategies for inhibiting polymerization, the root cause of the pathology. Because I have worked on normal and sickle hemoglobin for almost 50 years, my presentation will have a historical perspective and I will only consider those aspects of polymerization that have been closest to my own research. Accounts of polymerization and its role in disease pathogenesis can be found in previous articles by others.^2^, ^3^

## 2 PAULING'S DISCOVERY

According to his account,^4^, ^5^ Pauling instantly jumped to the conclusion that the disease was caused by an abnormal hemoglobin molecule in 1945 at dinner with a group of noted physicians. This was a committee appointed by President Franklin D. Roosevelt to recommend how the US government could assist in medical research at the conclusion of the second world war. At the dinner, William B. Castle, the famous Harvard hematologist, was telling the group about a disease called sickle cell anemia. He mentioned that the red cells have a characteristic sickle form when the cells are deoxygenated, but are prevented from sickling by oxygen. Pauling immediately pointed out that the “hemoglobin molecules in the red cells are involved and that [sickling] results from an abnormal kind of hemoglobin, which when deoxygenated has the power of combining with itself into long rigid rods that twist the red cell out of shape”.^4^ It might seem remarkable that a theorist, who showed how the new physics of quantum mechanics could explain chemical bonding, could jump immediately to this unprecedented conclusion about a disease with so little information. It is, however, not all that surprising; Pauling was one of the world's experts on hemoglobin at the time. He became interested in hemoglobin in the 1930's. In an incredibly brilliant paper, he presented a model that quantitatively explained cooperative oxygen binding with just two adjustable parameters.^6^ The fundamental thermodynamics of his model were rediscovered 31 years later and have been applied to many proteins.^7^ Pauling's sequential model was replaced in 1965 by the the allosteric model of Monod, Wyman, and Changeux (MWC). In the allosteric model cooperative oxygen binding results from a shift of the quaternary structural equilibrium from a low oxygen‐affinity **T** conformation, to a high oxygen‐affinity **R** conformation, as successive molecules of oxygen bind.^8^ As discussed below, the MWC model is extremely important for explaining how oxygen controls polymerization.

From 1945 to 1949, Pauling worked very hard with his post‐doctoral fellow, Harvey Itano, to find a difference in structure between hemoglobin A and hemoglobin S. The only structural tools available to them at the time were sedimenation and diffusion, from which they could obtain information on the size and shape of the two molecules and could find no difference. In 1948, Pauling visited Arne Tiselius who told him how to build an electrophoresis apparatus. With this apparatus Pauling discovered the difference between the two molecules, which he reported in his famous 1949 paper: “Sickle cell anemia, a molecular disease”.^1^ In the paper Pauling reported two major results. First, hemoglobin S is more positively charged than hemoglobin A and second, a 50‐50 mixture of hemoglobin S and hemoglobin A has a very similar electrophoretic pattern to the hemoglobin from the parents of sickle cell disease patients. From these two experimental facts, genius Pauling drew four grand conclusions. (i) Sickle cell anemia is due to an abnormal hemoglobin molecule‐ it is a “molecular disease.” (ii) From measurements of the acid‐base titration curves of the two molecules he determined that hemoglobin S has 2‐4 more net positive charges than hemoglobin A, most probably due to a difference in the number of charged amino acids. (iii) The mutation site must be on the molecular surface to cause aggregation and the structures of oxygenated and deoxygenated hemoglobin must be different to account for the fact that oxyhemoglobin S does not aggregate. (iv) The similarity of the electrophoretic patterns of the parent's hemoglobin and a 50‐50 mixture of hemoglobins A and S indicated that the disease was not autosomal dominant, as had previously been suggested, but followed an inheritance pattern of Mendelian genetics. Of course all of his conclusions turned out to be correct.^9^, ^10^, ^11^ What is not widely known is that Pauling effectively withdrew conclusions (ii) and (iv) in his presentation at the April 1950 meeting of the US National Academy of Sciences^12^ because no difference in charged amino acids could be detected using the technology of the time and hemoglobin S levels in sickle trait could be as low as 25%.^13^, ^14^ I have previously published a more extensive account of this very interesting part of the story.^15^

Pauling left three important questions for scientists ands clinicians to answer. What is the structure of the aggregated hemoglobin? ‐ his “long rigid rods.” What are the thermodynamics, kinetics, and mechanism of polymerization? How can the disease be treated?

## 3 THE POLYMER STRUCTURE

Structural studies began with a study by the Nobel Laureate Max Perutz in collaboration with his colleague John Finch at the MRC Laboratory of Molecular Biology in Cambridge. I received a preprint of the structure they published based on transmission electron microscopy. Their polymer was a hollow fiber consisting of a helical stack of rings of six hemoglobin S molecules with the 2‐fold symmetry axis of the hemoglobin molecule pointing radially.^16^ After making polarized optical absorption measurements on sickled cells, which gave information on the orientation of the hemoglobin molecules in the fiber,^17^ James Hofrichter and I went to Richard Feldmann at NIH, one of the pioneers in molecular graphics, to observe a three‐dimenional picture of hemoglobin and see how our results would add information to the fiber structure. To our surprise, the 2‐fold symmetry axis pointing radially required the site of the sickle mutation to face the solvent, far from any intermolecular contact. Consequently, there was no structural explanation on how the mutation could cause aggregation. I wrote a letter to Perutz, telling him that he made a mistake in his orientation of the molecule in the fiber, but that we could tilt the molecule sufficiently to allow a beta 6 intermolecular contact and still be consistent with his fiber structure and our optical data. Perutz replied with an invitation, with all expenses paid, to present our results at his January 1973 Royal Society meeting attended by all the hemoglobin luminaries of the day, including Martin Karplus, Robert Shulman, John Hopfield, Jeffries Wyman, Quentin Gibson, Eraldo Antonini and Maurizio Brunori. How many scientists today would invite an unkown young scientist (although he might have remembered me from the summer of 1962 when I worked with Sydney Brenner at the MRC lab in Cambridge) to their meeting to present a talk on what could be called his blunder?

The hollow structure was never seen again and was probably the fiber from some bacterial contaminant. The correct structure was discovered using transmission electron microscopy and sophisticated image reconstruction methods by Stuart Edelstein. Warner Love had solved the X‐ray structure of deoxyhemoglobin S a few years earlier, but it was not clear at the time whether his structure, which showed the details of a beta 6 intermolecular contact, had any relationship to the fibers that form in sickle cells.^18^ Edelstein recognized that he could construct a 14‐stranded fiber structure that is consistent with both fiber‐diffraction data of Magdoff‐Fairchild and our optically‐determined molecular orientation from seven helically twisted double strands of the X‐ray structure.^19^, ^20^ Subsequent polymerization studies by Ronald Nagel, Ruth Benesch, Rheinhold Benesch and their colleagues on mixtures of hemoglobin S with non‐S hemoglobin variants (site directed mutagenenis did not exist in those days) were critically important for additional structural studies (for key references see ref 21, 22). This information allowed Edelstein and Robert Josephs to build molecular models at amino acid residue resolution, which remain the currently operative models today.^21^, ^22^

## 4 THE THERMODYNAMICS

The thermodynamics of polymerization of completely deoxygenated, purified hemoglobin S turned out to be quite simple, but become considerably more complex when considering the control of polymerization by oxygen and mixtures of hemoglobin S with non‐S hemoglobins such as hemoglobins A and F. For sufficiently high concentrations, a solution of deoxyhemoglobin S becomes a viscous gel at equilibrium, that is, when no more polymer forms. As predicted by Allen Minton^23^ and confirmed experimentally by Ross et al.,^24^, ^25^ it is therefore very much like a crystal solution equilibrium, that is, a mixture of two phases, one solid and one liquid. In the case of hemoglobin S, the solid phase consists of aligned fibers, while the liquid phase contains only single hemoglobin molecules with no higher aggregates such as dimers or trimers.^26^ The two phase property was demonstrated from the observation that the concentration of hemoglobin S in the supernatant after sedimenting the fibers in an ultracentrifuge is independent of the total concentration of the sample.^24^, ^25^ As the total concentration increases, the amount of polymer increases, but the concentration in the liquid (supernatant) phase, the solubility of the fiber, remains unhanged. As the temperature increases the hydrophobic effect becomes stronger, so the solubility decreases because of the hydrophobic nature of the beta 6 contact in the fiber. The solubility has turned out to be a very accurate measure of the fiber stability.

Understanding how oxygen controls polymerization is the result of careful solubility studies, the direct measurement of binding of oxygen to the polymers, and the application of the MWC model. The solubility studies were first performed by Hofrichter using carbon monoxide as a surrogate for oxygen^27^ and later using oxygen by Sunshine et al^28^ In these studies application of linear dichroism allowed the measurement of the polymer binding curve of a gel, in which there is zero contribution to the spectral changes of the fiber as oxygen binds by molecules in the liquid phase because they are randomly oriented. The experiments of Sunshine et al. showed that polymer binding is non‐cooperative and that the solubility results are beautifully explained using the elegant polyphasic thermodynamic linkage relation of Gill and Wyman^29^ that relates the solubility to the binding curves of the polymer and liquid phases and includes activity coeffcients.^28^, ^30^ The activity coefficients, which multiply the measured concentration to give the thermodynamically effective concentration, are very large due to the geometric effect of excluded volume in the concentrated protein solutions. At 36 g/dL, for example, the activity coefficient is 100, so the solution behaves as if the concentration were 3,600 g/dL. Allen Minton and Philip Ross were primarily responsible for first recognizing the importance of activity coefficients.^25^, ^31^, ^32^

**Figure 1:**
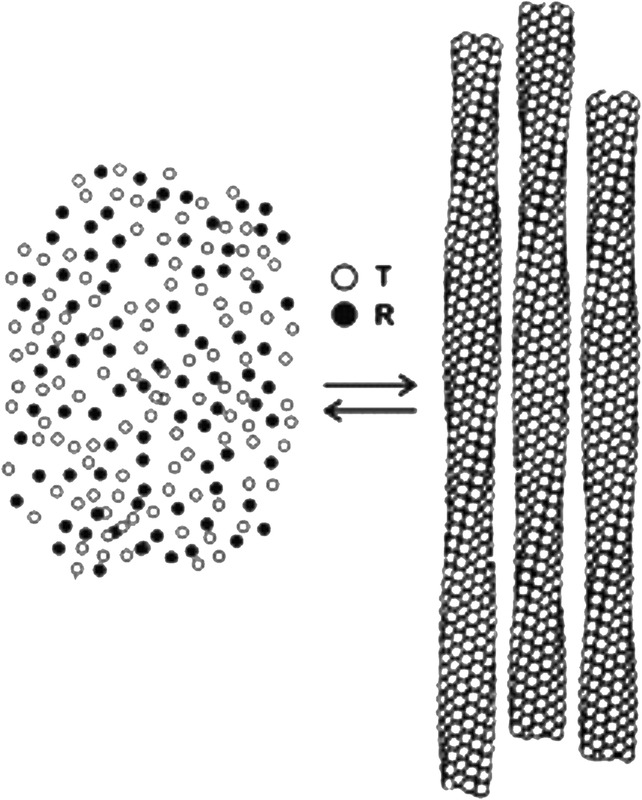
Schematic structure of Hb S gel partially saturated with oxygen showing that only the T quaternary structure enters the fiber. The total concentration of free HbS tetramers (left) is the solubility, which is an accurate measure of the thermodynamic stability of the fiber (right)

The solubility results are also almost quantitatively explained by postulating that only the T quaternary structure can enter the fiber (Figure 1),^28^ which is consistent with structural modeling by Eduardo Padlan.^33^ In fact, the MWC model does an impressive job, since there are no adjustable parameters in the calculation. An extension of the MWC model to include tertiary conformational changes^34^, ^35^ results in a significantly better quantitative explanation of the solubility data, albeit with one adjustable parameter (Henry et al., to be submitted to PNAS).

Understanding polymerization in mixtures of hemoglobin S with non‐hemoglobins S is much more complex. Because of their pathophysiological relevance, the most important mixtures are with hemoglobins A and F. The original theory on the co‐polymerization of non‐S hemoglobins was worked out by Allen Minton, but his mathematical formulation only applied to solutions in which the total deoxyhemoglobin concentration is equal to the solubility, a condition that is unachievable since polymerization takes an infinite amount of time near the zero polymer limit. For total concentrations larger then the solubility, that is, where the solution is supersaturated, the theoretical description becomes much more mathematically complicated. The equations are also more complicated^30^ because of the inclusion of activity coefficients resulting from the large excluded volume effects and explicitly including the activity of the aqueous solvent.^29^ Comparison of all of the data from many different laboratories has led to the conclusions that there is little or no co‐polymerization of the homotetramers α_2_β_2_^A^, a copolymerization probability for the hybrid α_2_β^A^β^S^ of about 0.4, and much less copolymerization of the hybrid α_2_γβ^S^. Tetramers that do not polymerize contribute significantly to determing the solubilty because they add to the excluded volume in the liquid phase and therefore increase the activity coefficients, which further increases the complexity of the theory.

There remain quite significant differences in copolymerization of hemoglobin F from different laboratories,^30^ which, given the importance of the role hemoglobin F today, should be repeated. Moreover, there is only one semi‐quantitative study on the solubility of S/F, S/A and S/A2 mixtures as a function of ligand saturation with carbon monoxide on hemolysates,^36^ so solubility data using purified hemoglobins and oxygen is needed to obtain more convincing data. The problem is that obtaining accurate solubility data for mixtures is technically demanding and requires a skilled experimentalist trained in physical chemistry or analytical biochemistry.

## 5 THE KINETICS AND MECHANISM

**Figure 2:**
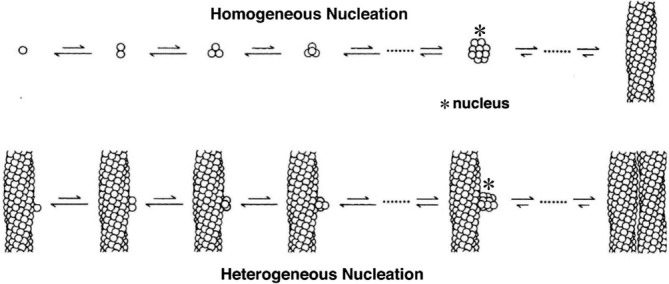
The double nucleation mechanism for sickle fiber formation.^47^ The basic idea of the mechanism is that there are two nucleated polymerization processes to sickle fiber formation, hence the name double nucleation mechanism.^40^, ^41^ The first fiber in any given volume forms by the classical Oosawa nucleation growth mechanism,^42^ in which there is a competition between the loss of translational and rotational entropy upon aggregation and the stability gained from intermolecular contacts, which is uphill in free energy until a critical nucleus is reached. Addition of molecules to the critical nucleus and all subsequent fiber growth is downhill in free energy. Formation of this first fiber is said to take place by homogeneous nucleation because it occurs in the bulk of the solution. Homogeneously nucleated fiber formation is quickly superceded by a secondary process, called heterogeneous nucleation, because fibers are nucleated on the surface of existing ones from which the growing nucleus gains additional stability from contacts with a fiber surface. As more fibers form there is increasing surface area for heterogeneous nucleation with the result that polymers grow exponentialy, which causes an apparent delay or what is often called a lag phase

The observation in slow temperature‐jump experiments of a delay prior to the appearance of polymers was almost simultaneously reported from three different laboratories in 1974 A delay period was observed by Hofrichter et al using birefringence and calorimetry,^37^ by Malfa and Steinhardt using viscosity,^38^ and by Moffat and Gibson using turbidity.^39^ A delay is also characteristic of the aggregation of peptides and proteins to form amyloid that is responsible for neurodegenerative diseases. The difference between amyloid formation and hemoglobin S polymerization is that there is a small concentration dependence to amyloid formation (about second ‐ fourth power), while there is an enormous concentration dependence (~30th power) to hemoglobin S polymerization.^37^ A double nucleation mechanism that could explain both the existence of a delay and a high concentration dependence was not developed until the work of Ferrone et al. in 1980 (Figure 2). This work used photodissociation of the carbon monoxide complex of hemoglobin S to create deoxyhemoglobin S at any temperature in milliseconds, instead of the tens of seconds to a minute in the earlier slow temperature jump experiments.^40^ This method also permitted the observation of sub‐second polymer formation on cells from patients with homozygous SS disease, which showed that there is little or no difference between deoxyhemoglobin S in purified solutions, and in red cells from patients with the disease.^43^

The double nucleation mechanism also accounts for the observation of large fluctuations in the delay time in small volumes as a result of the stochastic formation of the first homogenously‐nucleated fiber.^40^, ^41^, ^44^, ^45^ The experiment is similar in spirit to the first observation of single membrane channel opening and closings in patch clamp experiments, performed just a few years earlier, in which single protein channel kinetics is observed because of zillions of ions that flow when the channel is open to give rise to a measurable electrical current.^46^ In the sickle hemoglobin experiment, the zillions of fibers formed by the heterogeneous nucleation pathway that follow the formation of the first homogenously nucleated fiber in the volume could be detected by light scattering. The mechanism has stood the test of time.^47^ Interestingly, the double nucleation hemoglobin S polymerization mechanism is now being used to explain the aggregation kinetics of the Alzheimer's peptide.^48^, ^49^, ^50^, ^51^, ^52^

One of the most remarkable predictions of the double nucleation mechanism, confirmed by experiment, is that the concentration dependence of the rate of fiber formation by the homogeneous nucleation pathway should be approximately twice the concentration dependence of the delay time. Cao and Ferrone found a 50th power for the former and a 25th power for the latter,^45^ while Christoph et al. found 40th and 80th powers.^53^ Even though I would argue that an 80th power concentration dependence is one of the most amazing results in the history of chemical kinetics, the paper got the “grand slam.” That is, it was rejected without review by *Nature, Science, PNAS*, and *Cell*.

The most recent important discovery concerning hemoglobin S polymerization kinetics, is that the delay time can be obtained if the supersaturation is known, that is, the ratio of total hemoglobin concentration and the solubility, each multiplied by an activity coefficient, no matter what the composition of the solution ‐ partial oxygenation, mixtures of any kind, pH changes, etc.^54^ Since there is much more solubility data than than delay time data, this universal relation between delay time and supersaturation has become a valuable tool for theoretical calculations of sickling on any time scale^55^ (Henry et al, to be submitted to *PNAS*).

## 6 POLYMERIZATION AND DISEASE PATHOGENESIS

Given the recent large number of studies on processes other than polymerization, such as the role of adhesion, it has become a non‐trivial statement to say that the root cause of pathology in sickle cell disease is polymerization of hemoglobin. The discovery of the kinetics led to the hypothesis that the delay time relative to the transit time through the microcirculation is a major determinant of severity in sickle cell disease.^56^, ^57^ This concept synthesized a large amount of information on disease pathogenesis with a single postulate: factors that decrease the delay time or increase the transit time through the microcirculation, for example by increased adherence,^58^ increase clinical severity, while factors that increase the delay tme and shorten the transit time are associated with decreased clinical severity. Noguchi, Schechter and coworkers have proposed an alternative theory for pathogenesis that ignores the dynamics in which the amount of polymer formed at equilibrium is the key quantity.^2^ The dynamical scenario received strong support from the experiments of Mozzarelli et al., which showed that under in vivo conditions almost all cells would be sickled were polymerization at equilibrium,^59^ suggesting that patients would not survive once fetal hemoglobin was replaced by the abnormal adult hemoglobin S. The major deficiency in the Mozzarelli work is that sickling was determined on a non‐physiological time scale of minutes. Their conclusion, however, is strongly supported by updated calculations of in vivo sickling in which oxygen is dissociated from red cells on the in vivo seconds time scale, using measured intracellular hemoglobin concentration distributions^60^ (Henry et al, to be submitted to *PNAS*).

**Figure 3:**
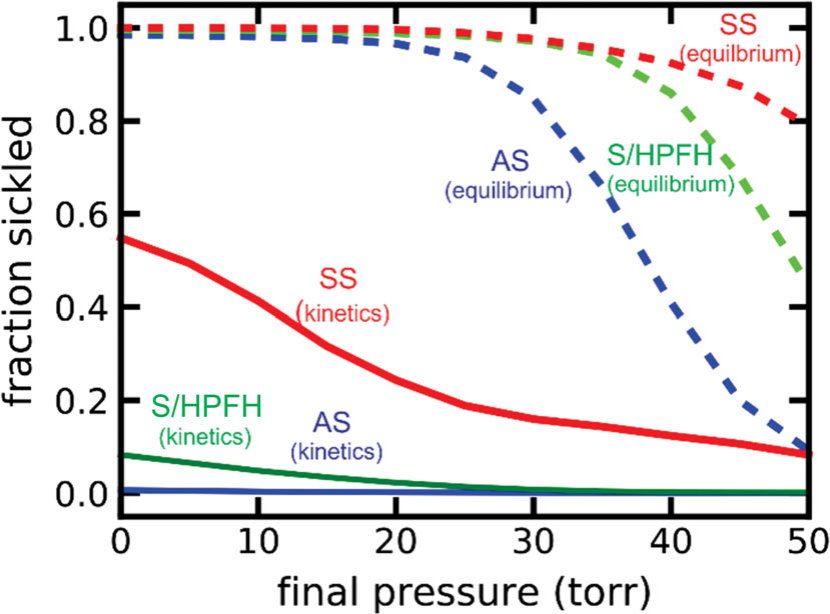
Sickling in vivo. Calclulation of in vivo sickling by Eric R. Henry and Troy Cellmer after a 2 second linear decrease of the oxygen pressure from 100 Torr oxygen pressure to various final oxygen pressures (continuous curves), and fraction of cells sickled if fiber formation were at equilibrium (ie, instantaneous polymerization), at oxygen pressures (dashed curves) for sickle cell anemia (SS),^60^ sickle cell disease with pancellular hereditary persistence of fetal hemoglobin (S/HPFH with 70%HbS, 30% HbF) and sickle cell trait (AS) (blue)

A sample of these calculations (Figure 3) show several interesting results. First, if polymerization were at equilibrium, almost every cell would contain fibers at partial pressures of oxygen in the tissues. The same is true for sickle cell disease with hereditary pancellular persistence of fetal hemoglobin (S/HPFH). Even in sickle trait, the vast majority of cells would contain fibers at tissue oxygen pressures between 10 Torr and 30 Torr. Consequently, it is the delay time, which allows the majority of cells to escape the narrowest vessels of the tissues before fibers form, a conclusion also reached in recent calculations by Ferrone,^60^ Karniadakis^61^ and their corworkers, that makes the disease survivable. This scenario is even more convincing for both S/HPFH and sickle trait because of the very little in vivo sickling, explaining why both are relative benign disorders. While equilibrium calculations are misleading as far as in vivo sickling is concerned, they do correlate with clinical severity because the delay time and the polymer fraction both depend on the supersaturation of the solutions. The difference is that the fraction polymerized depends on the first power of the supersaturation, while the delay time depends depends on the 30th power.^62^ Consequently, equilibrium calculations by Brittenham et al. are important in indicating that disease severity in the various sickle syndromes is determined by polymerization.^63^

### 6.1 Targeting polymerization for drug therapy

There are dramatic advances in curing sickle cell disease by hematopoetic stem cell transplantation,^64^ with gene therapy cures in the near future.^65^, ^66^ However, these treatments are expensive and require advanced medical facilities and are therefore not available to the vast majority of patients in the world suffering from sickle cell disease and may not be for decades. Consequently, what is urgently needed now is an inexpensive anti‐sickling pill. Therapy will not require a drug that completely inhibits sickling, but one that just increases the delay time to allow more cells to escape the microcirculation before fibers form. There is thus cause for optimism, as there are several mechanisms for increasing delay times other then by increasing fetal hemoglobin synthesis.^67^, ^68^, ^69^ These are described in detail in a recent *Blood* perspective,^70^ so they will only be mentioned here. They include increasing cell volume to decrease HbS concentration, binding a drug to the **R** conformation, thereby shifting the **T‐R** equilibrium toward the non‐polymerizing **R** conformation, decreasing intracellular 2,3‐diphosphoglycerate (2,3‐DPG) to destabilize the fiber and shifting the quaternary equilibrium toward **R,** increasing intracellular pH (2,3‐DPG also increases intracellular pH), and destabilizing the fiber by binding a drug to block intermolecular contacts.

Fortunately, there are now large libraries available for drug screening and sensitive high throughput screening methods that use intact cells.^71^ The most precious is the ReFrame library of the California Institute of Biomedical Research of the Scripps Institute that contains almost 12,000 compounds that have been tested in humans.^72^ I am therefore very optimistic that there will shortly be new anti‐sickling drugs in addition to hydroxyurea for treating sickle cell disease.

## References

[1] Pauling L , Itano HA , Singer SJ , Wells IC . Sickle cell anemia, a molecular disease. Science. 1949;110:543‐548. 15395398 10.1126/science.110.2865.543

[2] Noguchi CT , Schechter AN , Rodgers GP . Sickle cell disease pathophysiology. Baillieres Clin Haematol. 1993;6(1):57‐91. 8353318 10.1016/s0950-3536(05)80066-6

[3] Bunn HF . Mechanisms of disease ‐ pathogenesis and treatment of sickle cell disease. New Eng J Med. 1997;337(11):762‐769. 9287233 10.1056/NEJM199709113371107

[4] Pauling L . Abnormality of hemoglobin molecules in hereditary hemolytic anemias. Harvey Lect. 1954;49:216‐241. 13232548

[5] Pauling L . The normal hemoglobins and the hemoglobinopathies ‐ background. Texas Rep Biol Med. 1980;40:1‐7. 7034263

[6] Pauling L . The oxygen equilibrium of hemoglobin and its structural interpretation. Proc Natl Acad Sci U S A. 1935;21:186‐191. 16587956 10.1073/pnas.21.4.186 PMC1076562

[7] Koshland DE , Nemethy G , Filmer D . Comparison of experimental binding data and theoretical models in proteins containing subunits. Biochemistry. 1966;5(1):365‐385. 5938952 10.1021/bi00865a047

[8] Monod J , Wyman J , Changeux JP . On the nature of allosteric transitions: a plausible model. J Mol Biol. 1965;12(1):88‐118. 14343300 10.1016/s0022-2836(65)80285-6

[9] Ingram VM . Gene mutations in human haemoglobin ‐ chemical difference between normal and sickle cell haemoglobin. Nature. 1957;180(4581):326‐328. 13464827 10.1038/180326a0

[10] Muirhead H , Perutz MF . Structure of haemoglobin ‐ a 3‐dimensioanl fourier synthesis of reduced human haemoglobin at 5.5 A resolution. Nature. 1963;199(489):633‐638. 14074546 10.1038/199633a0

[11] Perutz MF , Watson HC , Muirhead H , Diamond R , Bolton W . Structure of haemoglobin ‐ X‐ray examination of reduced horse haemoglobin. Nature. 1964;203(494):687‐690. 14207261 10.1038/203687a0

[12] Pauling L , Itano HA , Wells IC . Sickle cell anemia hemoglobin. Science. 1950;111:459.

[13] Bunn HF , McDonald MJ . Electrostatic interactions in the assembly of hemoglobin. Nature. 1983;306(5942):498‐500. 6646230 10.1038/306498a0

[14] Huisman THJ . Percentages of abnormal hemoglobins in adults with heterozygosity for an alpha‐chain or beta‐chain variant. Amer J Hematol. 1983;14(4):393‐404. 6859036 10.1002/ajh.2830140411

[15] Eaton WA . Linus Pauling and sickle cell disease. Biophys Chem. 2003;100:109‐116. 12646356 10.1016/s0301-4622(02)00269-7

[16] Finch JT , Perutz MF , Bertles JF , Dobler J . Structures of sickled erythrocytes and of sickle cell hemoglobin fibers. Proc Natl Acad Sci USA. 1973;70(3):718‐722. 4123689 10.1073/pnas.70.3.718 PMC433343

[17] Hofrichter J , Hendricker DG , Eaton WA . Structure of hemoglobin S fibers. Optical determination of molecular orientation in sickled erythrocyrtes. Proc Natl Acad Sci U S A. 1973;70(12):3604‐3608. 4519649 10.1073/pnas.70.12.3604 PMC427289

[18] Wishner BC , Ward KB , Lattman EE , Love WE . Crystal structure of sickle cell deoxyhemoglobin S at 5 A resolution. J Mol Biol. 1975;98(1):179‐194. 1195378 10.1016/s0022-2836(75)80108-2

[19] Dykes GW , Crepeau RH , Edelstein SJ . Three‐dimensional reconstruction of the fibres of sickle cell haemoglobin. Nature. 1978;272:506‐510. 692655 10.1038/272506a0

[20] Dykes GW , Crepeau RH , Edelstein SJ . 3‐Dimenional reconstruction of the 14‐filament fibers of hemoglobin S. J Mol Biol. 1979;130(4):451‐472. 480359 10.1016/0022-2836(79)90434-0

[21] Carragher B , Bluemke DA , Gabriel B , Potel MJ , Josephs R . Structural analysis of polymers of sickle cell hemoglobin 1. sickle hemoglobin fibers. J Mol Biol. 1988;199(2):315‐331. 3351926 10.1016/0022-2836(88)90316-6

[22] Cretegny I , Edelstein SJ . Double strand packing in hemoglobin‐S fibers. J Mol Biol. 1993;230:733‐738. 8478930 10.1006/jmbi.1993.1195

[23] Minton AP . Thermodynamic model for gelation of sickle cell hemoglobin. J Mol Biol. 1974;82(4):483‐498. 4817792 10.1016/0022-2836(74)90243-5

[24] Ross PD , Hofrichter J , Eaton WA . Calorimetric and optical characterization of sickle cell hemoglobin gelation. J Mol Biol. 1975;96(2):239‐253. 1177306 10.1016/0022-2836(75)90345-9

[25] Ross PD , Hofrichter J , Eaton WA . Thermodynamics of gelation of gelation of sickle cell hemoglobin. J Mol Biol. 1977;115(2):111‐134. 22759 10.1016/0022-2836(77)90093-6

[26] Williams RC . Concerted formation of a gel of hemoglobin S. Proc Natl Acad Sci U S A. 1973;70(5):1506‐1508. 4514319 10.1073/pnas.70.5.1506 PMC433530

[27] Hofrichter J . Ligand binding and the gelation of sickle cell hemoglobin. J Mol Biol. 1979;128(3):335‐369. 439139 10.1016/0022-2836(79)90092-5

[28] Sunshine HR , Hofrichter J , Ferrone FA , Eaton WA . Oxygen binding by sickle‐cell hemoglobin polymers. J Mol Biol. 1982;158(2):251‐273. 7120411 10.1016/0022-2836(82)90432-6

[29] Gill SJ , Spokane R , Benedict RC , Fall K , Wyman J . Ligand‐linked phase equilibria of sickle cell hemoglobin. J Mol Biol. 1980;140:299‐312. 7431394 10.1016/0022-2836(80)90107-2

[30] Eaton WA , Hofrichter J . Sickle cell hemoglobin polymerization. Adv Prot Chem. 1990;40:63‐279. 10.1016/s0065-3233(08)60287-9 2195851

[31] Ross PD , Minton AP . Analysis of non‐ideal behavior in concentrated hemoglobin solutions. J Mol Biol. 1977;112(3):437‐452. 875025 10.1016/s0022-2836(77)80191-5

[32] Minton AP . Non‐ideality and thermodynamics of sickle cell hemoglobin gelation. J Mol Biol. 1977;110(1):89‐103. 845949 10.1016/s0022-2836(77)80100-9

[33] Padlan EA , Love WE . Refined crystal structure of deoxyhemoglobin S, 2. molecular interactions in the crystal. J Biol Chem. 1985;260(14):8280‐8291. 2409085

[34] Henry ER , Bettati S , Hofrichter J , Eaton WA . A tertiary two‐state allosteric model for hemoglobin. Biophys Chem. 2002;98(1–2):149‐164. 12128196 10.1016/s0301-4622(02)00091-1

[35] Henry ER , Mozzarelli A , Viappiani C , et al. Experiments on hemoglobin in single crystals and silica gels distinguish among allosteric models. Biophys J. 2015;109(6):1264‐1272. 26038112 10.1016/j.bpj.2015.04.037 PMC4576146

[36] Poillon WN , Kim BC , Rodgers GP , Noguchi CT , Schechter AN . Sparing effect of hemoglboin F and hemoglobin A2 on the polymerizatiio of hemoglobin S at physiological ligand saturations. Proc Natl Acad Sci U S A. 1993;90(11):5039‐5043. 7685112 10.1073/pnas.90.11.5039 PMC46649

[37] Hofrichter J , Ross PD , Eaton WA . Kinetics and mechanism of deoxyhemoglobin‐S gelation ‐ new approach to understanding sickle‐cell disease. Proc Natl Acad Sci U S A. 1974;71(12):4864‐4868. 4531026 10.1073/pnas.71.12.4864 PMC433999

[38] Malfa R , Steinhardt J . Temperature‐dependent latent period in the aggregation of sickle cell hemoglobin. Biochem Biophys Res Comm. 1974;59(3):887‐893. 4411783 10.1016/s0006-291x(74)80062-8

[39] Moffat K , Gibson QH . Rates of polymerization and depolymerization of sickle cell hemoglobin. Biochem Biophys Res Comm. 1974;61(1):237‐242. 4441395 10.1016/0006-291x(74)90558-0

[40] Ferrone FA , Hofrichter J , Sunshine HR , Eaton WA . Kinetic studies on photolysis induced gelation of sickle cell hemoglobin suggest a new mechanism. Biophys J. 1980;32:361‐377. 7248455 10.1016/S0006-3495(80)84962-9 PMC1327316

[41] Ferrone FA , Hofrichter J , Eaton WA . Kinetics of sickle hemoglobin polymerization 2. A double nucleation mechanism. J Mol Biol. 1985;183(4):611‐631. 4020873 10.1016/0022-2836(85)90175-5

[42] Ossawa F , Asakura S . The Thermodynamics of Protein Polymerization. New York: Academic Press; 1975.

[43] Coletta M , Hofrichter J , Ferrone FA , Eaton WA . Kinetics of sickle hemoglobin polymerization in single red‐cells. Nature. 1982;300(5888):194‐197. 7133139 10.1038/300194a0

[44] Hofrichter J . Kinetics of sickle hemoglobin polymerization 3. Nucleation rates determined from stochastic fluctuations in polymerization progress curves. J Mol Biol. 1986;189:553‐571. 3783684 10.1016/0022-2836(86)90324-4

[45] Cao ZQ , Ferrone FA . A 50th order reaction predicted and observed for sickle hemoglobin nucleation. J Mol Biol. 1996;256(2):219‐222. 8594190 10.1006/jmbi.1996.0079

[46] Neher E , Sakmann B . Single channel currents recorded from membrane of denervated frog muscle fibers. Nature. 1976;260(5554):799‐802. 1083489 10.1038/260799a0

[47] Ferrone FA . The delay time in sickle cell disease after 40 years: A paradigm assessed. Am. J. Hematol. 2015;90:438‐445. 25645011 10.1002/ajh.23958

[48] Cohen SIA , Vendruscolo M , Welland ME , Dobson CM , Terentjev EM , Knowles TPJ . Nucleated polymerization with secondary pathways. I. Time evolution of the principal moments. J Chem Phys. 2011;135(6):065105. 21842954 10.1063/1.3608916 PMC5017532

[49] Cohen SIA , Vendruscolo M , Dobson CM , Knowles TPJ . Nucleated polymerization with secondary pathways. II. Determination of self‐consistent solutions to growth processes described by non‐linear master equations. J Chem Phys. 2011;135(6):065106. 10.1063/1.3608917 PMC5036541 21842955

[50] Cohen SIA , Vendruscolo M , Dobson CM , Knowles TPJ . Nucleated polymerization with secondary pathways. III. Equilibrium behavior and oligomer populations. J Chem Phys. 2011;135(6):065107. 21842956 10.1063/1.3608918 PMC5017531

[51] Cohen SIA , Linse S , Luheshi LM , et al. Proliferation of amyloid‐beta 42 aggregates occurs through a secondary nucleation mechanism. Proc Natl Acad Sci U S A. 2013;110(24):9758‐9763. 23703910 10.1073/pnas.1218402110 PMC3683769

[52] Michaels TCT , Lazell HW , Arosio P , Knowles TPJ . Dynamics of protein aggregation and oligomer formation governed by secondary nucleation. J Chem Phys. 2015;143(5):054901. 26254664 10.1063/1.4927655

[53] Christoph GW , Hofrichter J , Eaton WA . Understanding the shape of sickled red cells. Biophys J. 2005;88(2):1371‐1376. 15542552 10.1529/biophysj.104.051250 PMC1305139

[54] Cellmer T , Ferrone FA , Eaton WA . Universality of supersaturation ratio in protein fiber formation. Nat Struct Mol Biol. 2016;23:459‐471. 27018803 10.1038/nsmb.3197 PMC7329141

[55] Dunkelberger EB , Metaferia B , Cellmer T , Henry ER . Theoretical simulation of red cell sickling upon deoxygenation based on the physical chemistry of sickle hemoglobin fiber formation. J Phys Chem B. 2018;122(49):11579‐11590. 30179501 10.1021/acs.jpcb.8b07638 PMC6422771

[56] Eaton WA , Hofrichter J , Ross PD . Delay time of gelation ‐ possible determinant of clinical severity in sickle‐cell disease. Blood. 1976;47(4):621‐627. 1260125

[57] Eaton WA , Hofrichter J . Hemoglobin‐S gelation and sickle‐cell disease. Blood. 1987;70(5):1245‐1266. 3311198

[58] Hebbel RP , Boogaerts MAB , Eaton JW , Steinberg MH . Erythrocyte adherence to endothelium in sickle cell anemia: a possible determinat of clinical severity. New Eng J Med. 1980;302(18):992‐995. 7366623 10.1056/NEJM198005013021803

[59] Mozzarelli A , Hofrichter J , Eaton WA . Delay time of hemoglobin‐S polymerization prevents most cells from sickling in vivo. Science. 1987;237(4814):500‐506. 3603036 10.1126/science.3603036

[60] Yosmanovich D , Rotter M , Aprelev A , Ferrone FA . Calibrating sickle cell disease. J Mol Biol. 2016;428(8):1506‐1514. 26975885 10.1016/j.jmb.2016.03.001

[61] Lu L , Li Z , Li H , Li XJ , Vekilov PG , Karniadakis GE . Quantitative prediction of erythrocyte sickling for the development of advanced sickle cell therapies. Sci Adv. 2019;5:eaax3905 10.1126/sciadv.aax3905 31457104 PMC6703859

[62] Hofrichter J , Ross PD , Eaton WA . Supersaturation in sickle cell hemoglobin solutions. Proc Natl Acad Sci U S A. 1976;73:3034‐3039. 10.1073/pnas.73.9.3035 PMC430918 9640

[63] Brittenham GM , Schechter AN , Noguchi CT . Hemoglobin S polymerization ‐ primary determinant of the hemolytic and clinical severity of the sickling syndormes. Blood. 1985;65(1):183‐189. 3965046

[64] Eapen M , Brazauskas R , Walters MC , et al. Effect of donor type and conditioning regimen intensity on allogeneic transplantation outcomes in patients with sickle cell disease: a retrospective multicentre, cohort study. Lancet Haematol. 2019;6:E585‐E596. 31495699 10.1016/S2352-3026(19)30154-1 PMC6813907

[65] Orkin SH , Bauer DE . Emerging genetic therapy for sickle cell disease. Ann Rev Med. 2019;70:257‐271. 30355263 10.1146/annurev-med-041817-125507

[66] Telen MJ , Malik P , Vercellotti GM . Therapeutic strategies for sickle cell disease: towards a multi‐agent approach. Nat Rev Drug Discov. 2019;18(2):139‐158. 30514970 10.1038/s41573-018-0003-2 PMC6645400

[67] Charache S , Terrin ML , Moore RD , et al. Effect of hydroxyurea on the frequency of painful crises in sickle cell anemia. N Engl J Med. 1995;332:1317‐1322. 7715639 10.1056/NEJM199505183322001

[68] Bridges KR , Barabino GD , Brugnara C , et al. A multiparameter analysis of sickle erythrocytes in patients undergoing hydroxyurea therapy. Blood. 1996;88(12):4701‐4710. 8977264

[69] Eaton WA , Hofrichter J . The biophysics of sickle cell hydroxyurea therapy. Science. 1995;268:1142‐1143. 7539154 10.1126/science.7539154

[70] Eaton WA , Bunn HF . Treatment of sickle cell disease by targeting Hb S polymerization. Blood. 2017;129(20):2719‐2726. 28385699 10.1182/blood-2017-02-765891 PMC5437829

[71] Dunkelberger EB , Metaferia B , Cellmer T , Henry ER . Theoretical simulation of red cell sickling upon deoxygenation based on the physical chemistry of Ssckle hemoglobin Fiber formation. J Phys Chem B. 2018;122(49):11579‐11590. 30179501 10.1021/acs.jpcb.8b07638 PMC6422771

[72] Janes J , Young ME , Chen EI . The ReFRAME library as a comprehensive drug repurposing library and its application to the treatment of cryptosporidiosis. Proce Natl Acad Sci USA. 2018;115:10750‐10755. 10.1073/pnas.1810137115 PMC6196526 30282735

